# Cardiac arrest events on Australian beaches

**DOI:** 10.1016/j.resplu.2025.101092

**Published:** 2025-09-09

**Authors:** David Reid, Kye Bostwick, Jasmin C. Lawes, Ogilvie Thom, Ned Douglas

**Affiliations:** a Surf Life Saving Australia, Bondi Beach, Australia; b School of Medical and Health Science, Edith Cowan University, Perth, Australia; c St John Ambulance (Western Australia), Perth, Australia; d Department of Critical Care, The University of Melbourne, Victoria, Australia; e College of Medicine and Dentistry, James Cook University, Townsville, Australia

**Keywords:** Resuscitation, Prehospital, out-of-hospital cardiac arrest (OHCA), Education, Simulation, Drowning

## Abstract

**Background:**

Surf Life Saving Australia (SLSA) members respond to cardiac arrests near their patrolling location and have successfully resuscitated many community members over the years. In addition, members often act as first responders to cardiac arrest events in community settings, workplaces or through applications such as GoodSAM®. The outcomes achieved are not known after an update to resuscitation guidelines.

**Objective:**

We aimed to analyse Surf Life Saving Australia data on cardiopulmonary resuscitation (CPR) and describe the epidemiology and outcomes of cardiac arrest on Australian beaches and factors associated with survival after CPR.

**Methods:**

Using SLSA’s national Incident Reporting Database held by Surf Life Saving Australia, anonymised incident report forms were analysed to determine the incidence, aetiology and outcomes of CPR events attended by SLSA members on Australian beaches.

**Results:**

Between December 2000 and May 2020 there were 158 incidents where CPR was provided SLSA members. Mean patient age was 46 years of age (SD 18.9), and 83 % were male. The majority (68 %) of patients were involved in water activities at the time of their requirement for CPR including swimming, wading, and watercraft activity.

Return of spontaneous circulation (ROSC) was achieved before transport to hospital in 34 (22 %) of cases. An automated external defibrillator (AED) was applied on 42 patients, with seven being defibrillated. The two variables associated with higher odds of survival were the incident occurring in the flagged area (OR 4.0 [95 % CI: 1.5–11.2]) and the use of oxygen therapy (OR 2.8 [95 % CI: 1.1–7.1]).

**Conclusion:**

Cardiac arrest at the beach is an uncommon event. Cardiac arrest close to patrolling location and the use of oxygen were associated with improved odds of ROSC.

## Highlights

• Between December 2000 and May 2020 CPR was provided Australian surf lifesavers at 158 beach incidents.
• Most patients were male (83 %) and involved in swimming, wading, and watercraft activities (68 %) prior to requiring CPR.
• Return of spontaneous circulation was achieved in 22 % of cases.
• Higher odds of survival were associated with the incident occurring in the flagged area and the use of oxygen therapy.
• A multicentre registry reporting consistent CPR data from beaches is required to establish if these associations are causal.

Approximately 26,000 out-of-hospital cardiac arrests (OHCA) occur in Australia each year,^1^ with only 13 % of patients surviving to return home.^2^ Variation in survival from OHCA can be explained by factors such as bystanders performing cardiopulmonary resuscitation (CPR) prior to ambulance arrival, the use of automated external defibrillators (AEDs), as well as time to ambulance dispatch and arrival.^3^ Many incidents on our coasts occur away from medical service, with the Surf Life Saving (SLS) movement existing as an important component of the first responder network along the coast. Australia benefits from the SLS movement, which is a nationally integrated surf lifesaving network comprising volunteer surf lifesavers and paid lifeguards, with the majority being volunteer surf lifesavers (herein collectively referred to as *lifesavers*.^4^, ^5^ This role responds to emergencies and provides assistance to beachgoers who need care or may be at risk, including responding to cardiac arrests and providing CPR.

Responding to cardiac arrest is an important role for lifesavers on the beach, but also often as first responders to cardiac arrest events in community settings, workplaces or through applications such as GoodSAM®. SLSA members were among the first lay rescuers in resuscitation in the 1960s.^6^ Lifesavers provide CPR in accordance with guidelines for community responders written by the Australian and New Zealand Committee on Resuscitation (ANZCOR),^7^ using AEDs and oxygen resuscitation equipment. In the three summer months of 2024–25 there were 51 coastal drowning deaths.^8^ However, non-drowning incidents also occur on Australian beaches,^9^ of which cardiac conditions have been identified as a causal factor in 52 % of deaths.^9^ Between 1973 and 1992 Surf Life Saving Queensland reported 171 resuscitations with a success rate of 67 %, particularly for those patients located close to the patrolled area.^10^ Between 1972 and 1983 SLSA reported 262 immersion victims at patrolled beaches of whom 162 (62 %) survived, 61 (23 %) of whom received expired air resuscitation or and 29 (11 %) chest compressions.^11^ These findings came from studies conducted prior to a major update to CPR training that occurred in 2000, where SLSA changed many of the technical details and introduced automated external defibrillators (AEDs) to practice. Whether survival has improved due to SLSA practice since the new guidelines were released is unknown, and survival in other settings has noticeably improved.^12^

SLSA wished to increase survival after cardiac arrest and was considering implementing a resuscitation quality improvement program as one potentially promising intervention.^13^ Prior to implementation of the program, SLSA sought to understand the epidemiology and outcomes from cardiac arrest on Australian beaches to inform the education intervention. This epidemiological study aims to describe outcomes from cardiac arrests attended by lifesavers in Australia and identify predictive factors associated with achieving the return of spontaneous circulation.

## Methods

We conducted a retrospective cohort study of patients who received CPR from surf lifesavers across Australian beaches in all states and territories between December 2000 and May 2020. We used the strengthening the reporting of observational studies in epidemiology (STROBE) checklist^14^ to ensure that all relevant components of the study were reported. The Edith Cowan University Human Research Ethics Committee approved the project (2023-04183-REID). We conducted the study in accordance with the Declaration of Helsinki.^15^

### Population

**Fig. 1:**
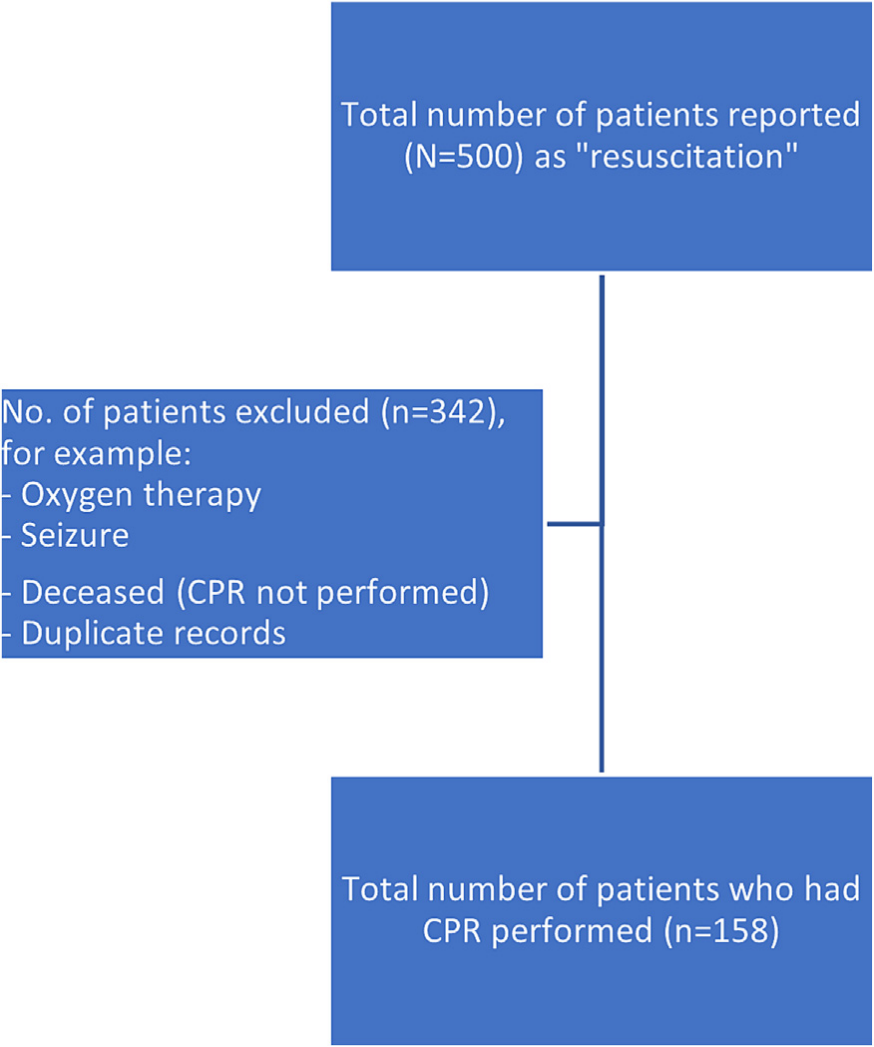
STROBE diagram.

We included patients who were identified by Australian surf lifesavers as unconscious, not breathing normally, and who received CPR and where the resuscitation attempt was recorded in the SLSA national incident report database (IRD). We excluded cases where CPR was not performed, including the application of oxygen therapy in the conscious patient, seizures or where CPR was not performed on an obviously deceased patient (Fig. 1).

### Aims, objectives, exposures and outcomes

We aimed to describe outcomes from cardiac arrests attended by lifesavers in Australia and identify predictive factors associated with achieving the return of spontaneous circulation. The primary objective was to describe the rate of return on spontaneous circulation (ROSC) achieved before ambulance transport to hospital, and the primary outcome was the incidence of ROSC before ambulance transport to hospital or termination of resuscitation.

The secondary objective was to explore whether factors including patient age or sex, location of resuscitation, delay until ambulance arrival (defined using the organisation’s existing categories) and the provision of oxygen or an AED was associated with higher odds of survival. These factors constituted the exposures of interest potentially associated with the outcome of ROSC.

### Data management

Lifesavers who treated the patient entered incident data on a standardised paper or electronic incident report form after a CPR event had occurred. An example form is shown in Appendix 1. Paper forms were transcribed into electronic format by organisation staff. Data was then collated into a national database of first aid forms, which is maintained and owned by Surf Life Saving Australia.

We initially selected all incidents in which an ambulance was requested in order to maximise the efficiency of the search for records, and then selected all records where any of the terms “CPR”, “resuscitation”, “AED” or “BVM” were found in database fields labelled “incident description”, “cause of incident” or “initial treatment provided”. A single author (KB) then reviewed the incident description field to ensure that the incident involved a patient provided CPR by lifesavers.

A single author (KB) reviewed the incident description to extract exposure variables such as age (defined as patient age on the day of the incident), location of incident, distance from the SLSA patrolled area (dichotomised as inside the flags vs not inside the flags), the use of oxygen (defined as oxygen being recorded as being used during the resuscitation) and AED use (defined as an AED being applied during the resuscitation by a lifesaver), as well as the number of shocks delivered. Where uncertainty in the data existed a second author (DR or ND) reviewed the case before it was included.

### Sample selection and statistical analysis

We used a convenience sample of all available records from the study period to maximise the precision of the estimates and reduce the risk of selection bias. We present the primary outcome descriptively, with a proportion and 95 % confidence interval.

We investigated associations between increasing age, AED and oxygen use and successful return of spontaneous circulation using logistic regression. We examined unadjusted odds ratios and included all potential prediction variables. All analyses were performed in Stata 17 (College Station, USA).

## Results

There were 158 CPR events on Australian beaches identified from within the initial data set of 500 incident reports coded in the IRD as “resuscitation” (Fig. 1).

**Fig. 2:**
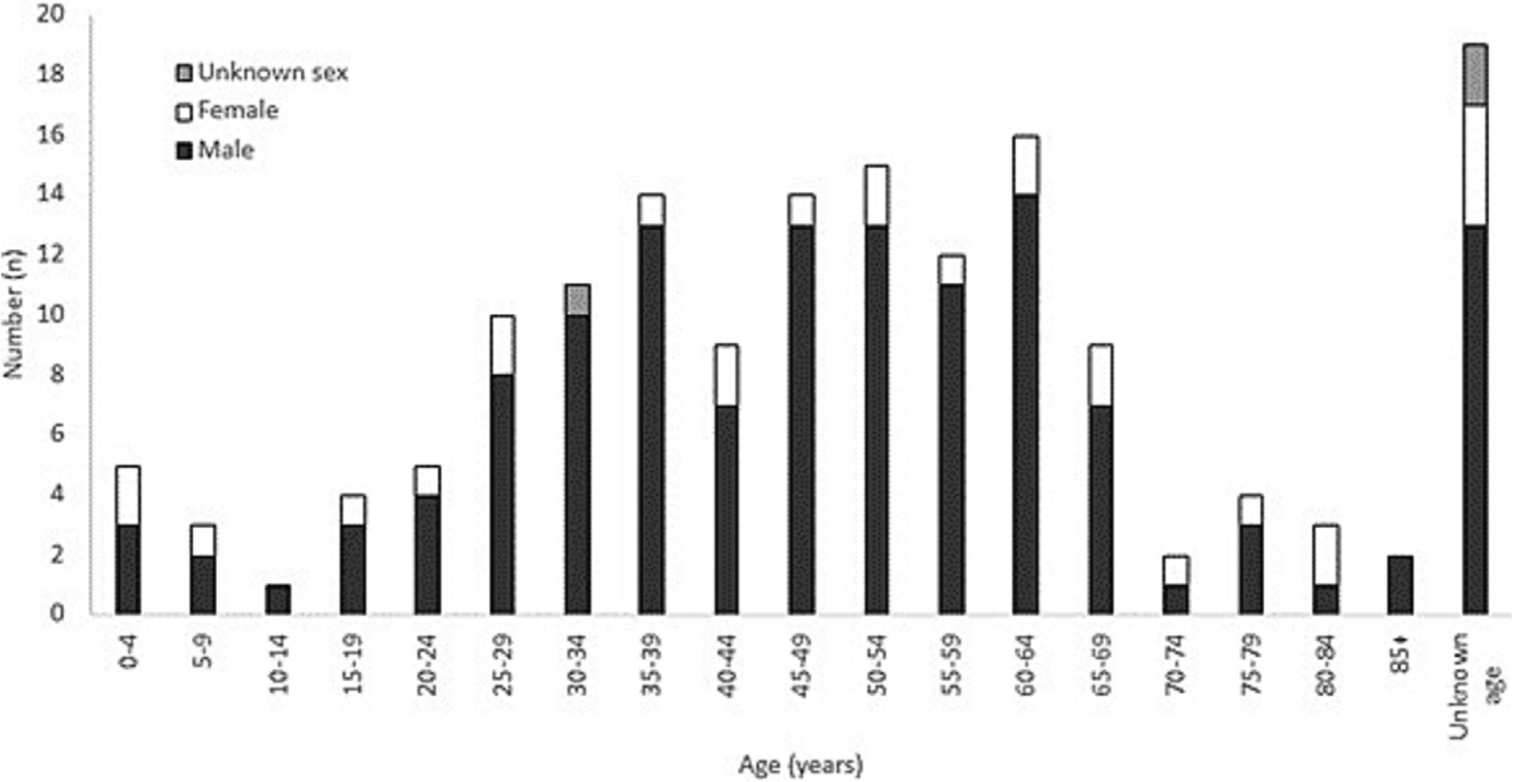
Reported age and sex of patients who received CPR by SLSA members during the study period. Full demographic and exposure details are presented in Table 1, Table 2 respectively.

The median patient age (n = 139) was 46 years (SD = 18.9), and full details of the age distribution are shown in Fig. 2. Of the 155 events in which patient sex was recorded, 83 % (n = 129) were male and 17 % (n = 26) female (Fig. 2). Descriptive analsyes are shown in Table 2.

**Table 1:** Baseline characteristics.

**Parameter**	**Median or n (%)**	**IQR or 95 % CI**
Age (years)	46	27 (33–60)
Sex (n, %)		
- Male	129 (83 %)	75–87 %
- Female	26 (17 %)	11–23 %

**Table 2:** Exposure variables.

**Parameter**	**N**	**%**	**95 % CI**
***Activity at time of event***			
Swimming or wading	84	53 %	46–62 %
On land	46	30 %	22–37 %
On rocks	4	3 %	1–6 %
Using watercraft	13	8 %	5–14 %
Other activity in water	9	5 %	3–11 %
Unknown	2	1 %	0–5 %
***Distance from patrolled location***			
Within flagged area	19	12 %	10–25 %
Immediately adjacent to flagged area	11	7 %	5–17 %
Within 1 km of flagged area	41	26 %	17–46 %
More than 1 km from flagged area	32	20 %	20–38 %
Unknown	55	35 %	27–43 %
***AED use***			
AED used	42	27 %	19–34 %
AED shock delivered	7	4 %	2–9 %
- One shock	3	2 %	0–5 %
- Two shocks	3	2 %	0–5 %
- Three or more shocks	1	0.6 %	0–3 %
AED not used	3	2 %	0–5 %
Unknown	113	72 %	64–78 %
***Oxygen***			
Oxygen used	25	16 %	11–22 %
Oxygen not used	2	1 %	0–5 %
Oxygen status unknown	131	83 %	76–88 %
***Time to ambulance arrival***			
<1 min	2	1 %	0–5 %
1–3 min	2	1 %	0–5 %
3–5 min	7	4 %	2–9 %
5–10 min	37	23 %	17–31 %
>10 min	34	22 %	15–29 %
Unknown	76	48 %	40–56 %

Of the patients who received CPR, 34 (22 %, 95 % CI: 17 %-31 %) achieved ROSC. A further 39 (25 %, 95 % CI: 18–32 %) were declared deceased at the scene and 16 (10 %, 95 % CI 6–16 %) were deceased at hospital without achieving ROSC, while 66 (42 %, 95 % CI: 34–50 %) cases had an unknown outcome.

We constructed logistic regression models for the outcome of ROSC, using the potential predictor variables of age, female sex, the incident occurring in the flagged patrol area, the estimated delay until ambulance arrival in categories of <1 min, 1–3 min, 3–5 min, 5–10 min and >10 min, AED use and oxygen use using a complete case analysis. Unadjusted odds ratios and p values are reported in Table 3. The two variables associated with higher odds of survival were the incident occurring in the flagged area (OR 4.61 [95 % CI: 1.71–12.5], p < 0.003) and the use of oxygen therapy (OR 3.23 [95 % CI: 1.31–7.94], p < 0.01). All other variables were not significantly associated with higher odds of survival (p > 0.05; Table 3).

**Table 3:** Unadjusted odds ratios for return of spontaneous circulation.

	**Unadjusted**		
**Parameter**	**OR**	**95 % Confidence interval**	**P value**
Age (years)	0.99	0.98–1.01	0.32
Sex (female)	1.24	0.48–3.23	0.66
Incident occurs in patrolled area (yes)	4.61	1.71–12.5	0.003*
Time to ambulance arrival (category)	1.06	0.89–1.26	0.483
Use of oxygen (yes)	3.23	1.31–7.94	0.01*
Use of AED (yes)	1.72	0,78–3.8	0.18

* = statistically significant at p < 0.05.

## Discussion

This study presents an updated estimate of the survival after CPR on Australian beaches after the deployment of modern CPR guidelines. This study found a lower number of successful resuscitations (23 % ROSC) than the previous Queensland study which reported a rate of achieving ROSC of 67 %,^10^ although this may be due to a change of definition of resuscitation between the two time points. In a comparable national study between 1972 and 1983, 62 % survived resuscitation,^11^ however not all patients in those cohorts received chest compressions and a significant proportion may not have been in cardiac arrest as patients could be included in these data simply because they were provided oxygen. Our results imply an opportunity to improve survival after cardiac arrest on Australian beaches.

Our data illustrated that the majority of surf lifesaver CPR responses are for patients who have likely drowned with probable immersion impacts, based on their activity at time of cardiac arrest (54 % swimming or wading). The rate of ROSC achieved in this study (22 %) is commensurate with that found across out-of-hospital cardiac arrests, which ranges from 20 % to over 50 %.^16^ In certain settings, such as the Melbourne Cricket Ground, survival is much higher, with 86 % leaving the venue with ROSC and 71 % being discharged home from hospital in one study.^17^ The difference in outcome in our study may be explained by different pathology, with cardiac arrest due to drowning potentially being more difficult to resuscitate due to more lethal conditions such as pulmonary oedema or aspiration, than cardiac arrest due to coronary artery disease.

Our exploratory analysis suggested several interesting findings that could be the subject of prospective studies, including higher odds of ROSC when a CPR event occurred within the area patrolled by lifesavers. This finding could be explained by faster response times, and either a shorter submersion time or a faster recognition of need and commencement of CPR coupled with more rapid provision of oxygen. This implies a bystander CPR effect in drowning resuscitation, reaffirming the WHO’s call to promote CPR training of bystanders.^18^ Similarly, the increased odds of survival when oxygen is provided during CPR after adjusting for incident location is also an important finding that supports current practice and is a novel finding to our knowledge. A recent review found no evidence in favour of oxygen during CPR for drowning^19^ and a subsequent study found limited improvement in oxygen saturation in drowning patients being treated with oxygen.^20^ However, there is some evidence that CPR including ventilation after drowning is associated with a higher likelihood of recovery compared to compression only CPR^21^, ^22^ and our data is consistent with that finding. The potential impact of oxygen in drowning associated cardiac arrest lies in the differing pathophysiology when compared to primary circulatory arrest, specifically that anoxia leads to cardiac arrest in drowning and there is substantial face validity to replenishing oxygen as quickly as possible.^22^ Our finding that AED use was not associated with ROSC is in contrast to previous research primarily recruiting patients in shockable rhythms.^23^ AED use is associated with nearly double the survival rate of an OHCA when compared to non-AED CPR in other settings^24^ outside of drowning cohorts. Our data suggests that AED use may only be useful in our setting when the patient has suffered a cardiac arrest from a different pathology and is in a shockable rhythm.^25^ Our results did not reveal an effect of sex on ROSC after CPR, suggesting that the higher risk among males aligns more with increased participation and the higher drowning rates observed among men^8^, ^9^, ^26^, ^27^ rather than treatment effects, reinforcing the need for continued prevention efforts and broader risk mitigation in this high-risk cohort prior to cardiac arrest.^8^

### Limitations

The study has several limitations. Firstly, the generalisability of the results may be limited as the response and treatment arrangements are specific to SLSA. Incomplete and missing data was common due to the non-healthcare background of most surf lifesavers, contributing to the wide confidence intervals seen in the effect size estimates and consequent uncertainty in both the outcomes observed and the treatment effects noted. Furthermore, the relatively low exposure and event rates may mean that the secondary outcome analysis is underpowered to detect important clinical effects. Additionally, this data included both drowning and non-drowning causes of cardiac arrest, and this may contribute to heterogeneity of treatment effects. This data does not include long-term follow-up of patients, and further research is required to determine the long-term neurological outcomes of patients, including survival to hospital discharge and degree of impairment after discharge. In common with all retrospective research there is likely to be significant unmeasured confounding in the results and causality between exposures and outcomes cannot be inferred.

The interpretation of treatment effects in our logistic regression modelling was difficult due to missing data and potential correlation between elements of treatment and other confounding variables.

## Conclusions

CPR provided by lifesavers on Australian beaches was associated with a 24 % return of spontaneous circulation rate, which may be explained by drowning being the most common pathology causing cardiac arrest in this patient cohort. A CPR incident occurring in the patrolled area, and the administration of oxygen during CPR was associated with a higher odds of patient survival. These results highlight an opportunity to further monitor and track cardiac arrest events and conduct prospective research to inform resuscitation practices on Australian beaches.

## CRediT authorship contribution statement

**David Reid:** Writing – review & editing, Writing – original draft, Validation, Supervision, Project administration, Methodology, Investigation, Formal analysis, Conceptualization. **Kye Bostwick:** Writing – review & editing, Investigation, Formal analysis, Data curation. **Jasmin C. Lawes:** Writing – review & editing, Investigation, Formal analysis. **Ogilvie Thom:** Writing – review & editing, Formal analysis. **Ned Douglas:** Writing – review & editing, Validation, Methodology, Investigation, Formal analysis, Data curation, Conceptualization.

## Funding

No funding was received for this study.

## Declaration of competing interest

The authors declare that they have no known competing financial interests or personal relationships that could have appeared to influence the work reported in this paper.
