# Supplementary material for: Cardiac arrest events on Australian beaches
Source: Resusc Plus. 2025 Sep 9;26:101092. doi: 10.1016/j.resplu.2025.101092 (PMC12495224; doi:10.1016/j.resplu.2025.101092)
Supplement: Supplementary Data 1 [file mmc1.docx]

**Appendix 1 – Data collection form**


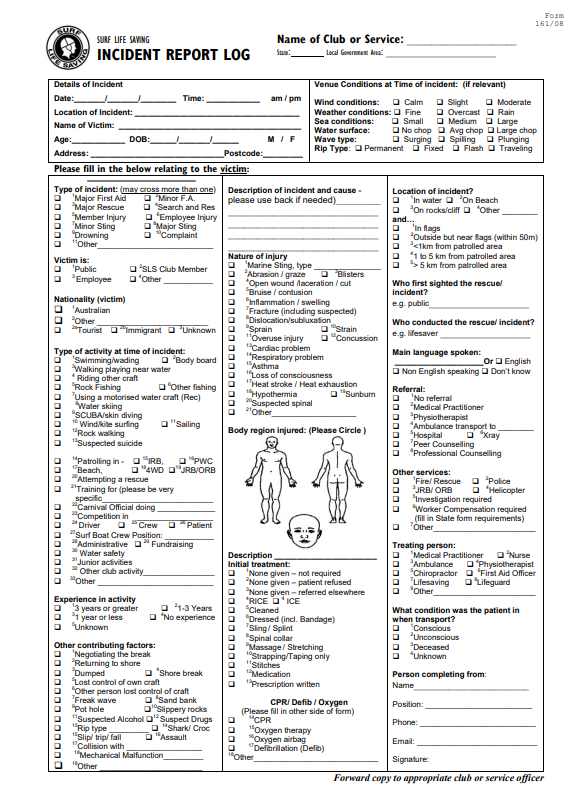


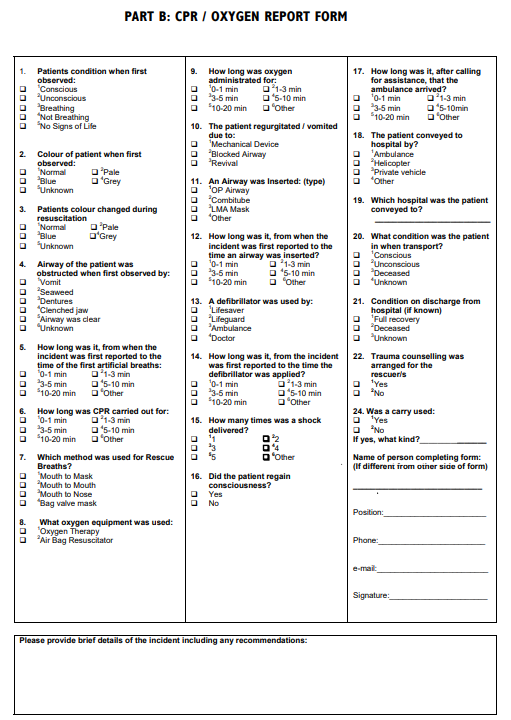


**Appendix 2 – Model dianostics for the logistic regression model**

***Directed acyclic graph***


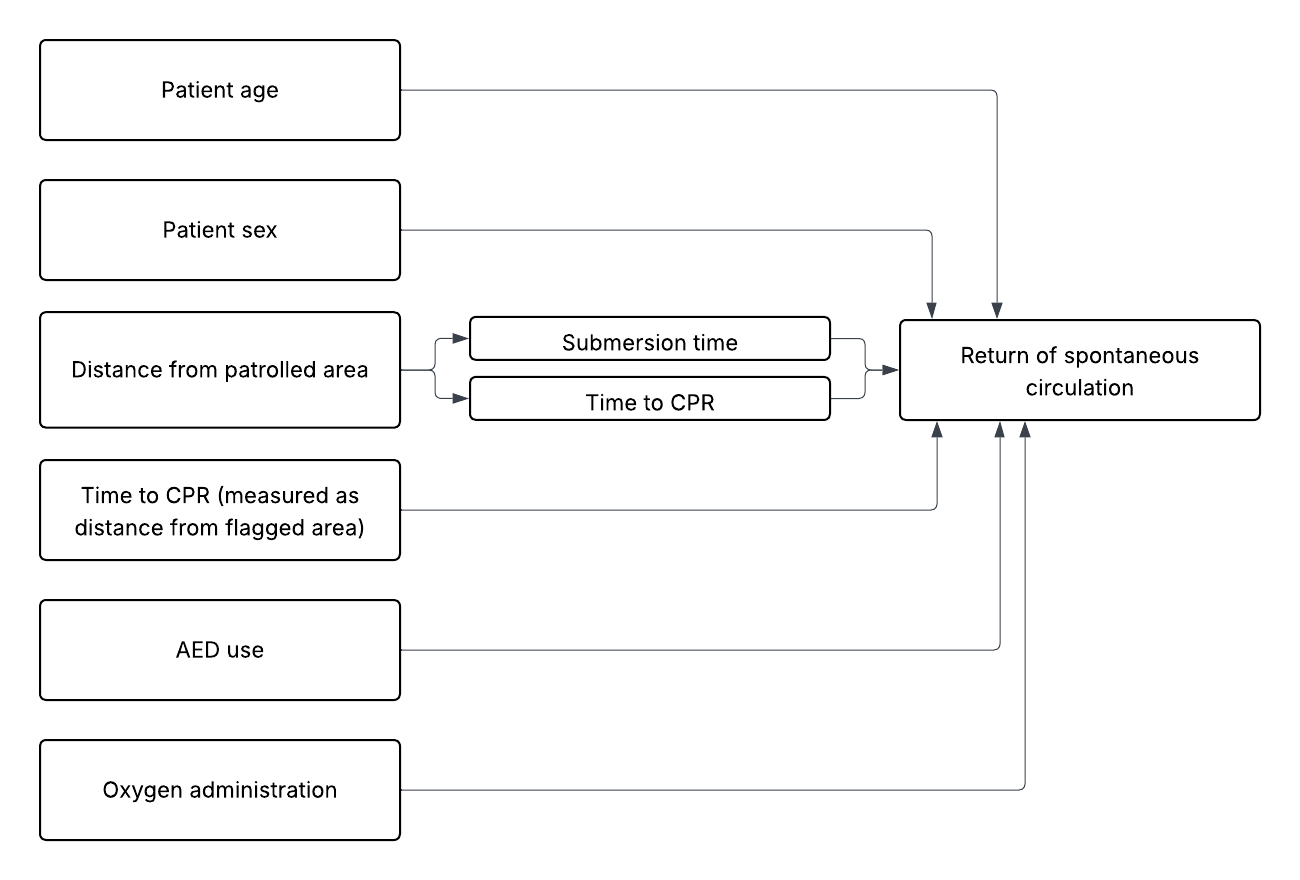


***Classification***

Logistic model for ROSC

-------- True --------

Classified | D ~D | Total

---------------------+-------------------------------+-----------

+ | 4 2 | 6

- | 33 116 | 149

---------------------+-------------------------------+-----------

Total | 37 118 | 155

---------------------+-------------------------------+-----------

Sensitivity Pr( +| D) 10.81%

Specificity Pr( -|~D) 98.31%

Positive predictive value Pr( D| +) 66.67%

Negative predictive value Pr(~D| -) 77.85%

---------------------+-------------------------------+-----------

False + rate for true ~D Pr( +|~D) 1.69%

False - rate for true D Pr( -| D) 89.19%

False + rate for classified + Pr(~D| +) 33.33%

False - rate for classified - Pr( D| -) 22.15%

---------------------+-------------------------------+-----------

Correctly classified 77.42%

---------------------+-------------------------------+----------

***Correlation coefficients between potential predictor variables***

| ROSC

e(V) | Pati~Age Female InFlags AmbDelay Oxygen AED _cons

-------------+----------------------------------------------------------------------

ROSC |

PatientsAge | 1.0000

Female | 0.0492 1.0000

InFlags | -0.0890 -0.1024 1.0000

AmbDelay | -0.0721 0.0320 -0.0881 1.0000

Oxygen | 0.1702 0.1110 -0.0357 -0.1762 1.0000

AED | -0.1880 -0.1865 -0.0195 -0.1771 -0.4565 1.0000

_cons | -0.7245 -0.2071 -0.0570 -0.3053 -0.1819 0.0515 1.0000

***Variance inflation factors for predictor variables***

Baseline model

Variable | VIF 1/VIF

-------------+----------------------

AED | 1.37 0.729388

Oxygen | 1.34 0.746628

AmbDelay | 1.11 0.902172

PatientsAge | 1.06 0.946161

InFlags | 1.05 0.954225

Female | 1.05 0.956732

-------------+----------------------

Mean VIF | 1.16

Pared model

Variable | VIF 1/VIF

-------------+----------------------

InFlags | 1.03 0.974522

Oxygen | 1.03 0.974522

-------------+----------------------

Mean VIF | 1.03
